# First results on survival from a large Phase 3 clinical trial of an autologous dendritic cell vaccine in newly diagnosed glioblastoma

**DOI:** 10.1186/s12967-018-1507-6

**Published:** 2018-05-29

**Authors:** Linda M. Liau, Keyoumars Ashkan, David D. Tran, Jian L. Campian, John E. Trusheim, Charles S. Cobbs, Jason A. Heth, Michael Salacz, Sarah Taylor, Stacy D. D’Andre, Fabio M. Iwamoto, Edward J. Dropcho, Yaron A. Moshel, Kevin A. Walter, Clement P. Pillainayagam, Robert Aiken, Rekha Chaudhary, Samuel A. Goldlust, Daniela A. Bota, Paul Duic, Jai Grewal, Heinrich Elinzano, Steven A. Toms, Kevin O. Lillehei, Tom Mikkelsen, Tobias Walbert, Steven R. Abram, Andrew J. Brenner, Steven Brem, Matthew G. Ewend, Simon Khagi, Jana Portnow, Lyndon J. Kim, William G. Loudon, Reid C. Thompson, David E. Avigan, Karen L. Fink, Francois J. Geoffroy, Scott Lindhorst, Jose Lutzky, Andrew E. Sloan, Gabriele Schackert, Dietmar Krex, Hans-Jorg Meisel, Julian Wu, Raphael P. Davis, Christopher Duma, Arnold B. Etame, David Mathieu, Santosh Kesari, David Piccioni, Manfred Westphal, David S. Baskin, Pamela Z. New, Michel Lacroix, Sven-Axel May, Timothy J. Pluard, Victor Tse, Richard M. Green, John L. Villano, Michael Pearlman, Kevin Petrecca, Michael Schulder, Lynne P. Taylor, Anthony E. Maida, Robert M. Prins, Timothy F. Cloughesy, Paul Mulholland, Marnix L. Bosch

**Affiliations:** 1 University of California Los Angeles (UCLA) David Geffen School of Medicine & Jonsson Comprehensive Cancer Center, Los Angeles, CA USA; 2 King’s College London School of Medical Education, London, UK; 3 University of Florida, Gainesville, FL USA; 4 Washington University, St. Louis, MO USA; 5 Abbott Northwestern Hospital, Minneapolis, MN USA; 6 Swedish Medical Center, Swedish Neuroscience Institute, Seattle, WA USA; 7 University of Michigan Medical School, Ann Arbor, MI USA; 8 University of Kansas Cancer Center, Kansas City, KS USA; 9 Sutter Institute for Medical Research, Sacramento, CA USA; 10 Columbia University Medical Center, New York, NY USA; 11 Indiana University Simon Cancer Center, Indianapolis, IN USA; 12 Overlook Medical Center, Summit, NJ USA; 13 University of Rochester Medical Center, Rochester, NY USA; 14 Rush University Medical Center, Rochester, USA; 15 Rutgers Cancer Institute, New Brunswick, NJ USA; 16 University of Cincinnati Medical Center, Cincinnati, OH USA; 17 Hackensack University Medical Center, Hackensack, NJ USA; 18 UC Irvine Medical Center, Irvine, CA USA; 19 Winthrop-University Hospital, Mineola, NY USA; 20 Rhode Island Hospital, Providence, RI USA; 21 University of Colorado Hospital, Aurora, CO USA; 22 Henry Ford Health System, Detroit, MI USA; 23 St. Thomas Research Institute, Nashville, TN USA; 24 University of Texas Health Science Center, San Antonio, TX USA; 25 University of Pennsylvania Perelman School of Medicine, Philadelphia, PA USA; 26 University of North Carolina, Chapel Hill, NC USA; 27 City of Hope National Medical Center, Duarte, CA USA; 28 Thomas Jefferson University, Philadelphia, PA USA; 29 St. Joseph Hospital, Newport Beach, CA USA; 30 Vanderbilt University, Nashville, TN USA; 31 Beth Israel Deaconess Medical Center, Boston, MA USA; 32 Baylor University Medical Center, Dallas, TX USA; 33 Illinois CancerCare, Peoria, IL USA; 34 Medical University of South Carolina, Charleston, SC USA; 35 Mount Sinai Comprehensive Cancer Center, Miami, FL USA; 36 University Hospitals Case Medical Center, Cleveland, OH USA; 37 University Hospital Carl-Gustav-Carus of Technical University, Dresden, Germany; 38 BG-Klinikum Bergmannstrost, Halle, Germany; 39 Tufts University School of Medicine, Boston, MA USA; 40 Stony Brook University, Stony Brook, NY USA; 41 Hoag Cancer Center, Newport Beach, CA USA; 42 H. Lee Moffit Cancer Center and Research Institute, Tampa, FL USA; 43 CHUS-Hopital Fleurimont, Sherbrooke University, Sherbrooke, QC Canada; 44 UCSD Health System, UC San Diego, San Diego, CA USA; 45 Neurochirurgische Klinik University Clinic Hamburg-Eppendorf, Hamburg, Germany; 46 Houston Methodist, Houston, TX USA; 47 Geisinger Health System, Danville, PA USA; 48 Klinikum Chemnitz GGMBH, Chemnitz, Germany; 49 Saint Luke’s Cancer Institute, Kansas City, MO USA; 50 Kaiser Permanente Northern California, Redwood City, CA USA; 51 Kaiser Permanente Southern California, Los Angeles, CA USA; 52 University of Kentucky College of Medicine, Lexington, KY USA; 53 Colorado Neurological Institute, Englewood, CO USA; 54 Montreal Neurological Institute and Hospital, McGill University, Montreal, Canada; 55 Northwell Hofstra School of Medicine, Lake Success, NY USA; 56 Department of Neurology, Alvord Brain Tumor Center, University of Washington, Seattle, WA USA; 57 University College Hospitals, London, UK; 58 Northwest Biotherapeutics Inc., Bethesda, MD USA; 59 NYU Winthrop Hospital, Mineola, NY USA

**Keywords:** Glioblastoma, Immunotherapy, Dendritic cell, Vaccine

## Abstract

**Background:**

Standard therapy for glioblastoma includes surgery, radiotherapy, and temozolomide. This Phase 3 trial evaluates the addition of an autologous tumor lysate-pulsed dendritic cell vaccine (DCVax^®^-L) to standard therapy for newly diagnosed glioblastoma.

**Methods:**

After surgery and chemoradiotherapy, patients were randomized (2:1) to receive temozolomide plus DCVax-L (n = 232) or temozolomide and placebo (n = 99). Following recurrence, all patients were allowed to receive DCVax-L, without unblinding. The primary endpoint was progression free survival (PFS); the secondary endpoint was overall survival (OS).

**Results:**

For the intent-to-treat (ITT) population (n = 331), median OS (mOS) was 23.1 months from surgery. Because of the cross-over trial design, nearly 90% of the ITT population received DCVax-L. For patients with methylated MGMT (n = 131), mOS was 34.7 months from surgery, with a 3-year survival of 46.4%. As of this analysis, 223 patients are ≥ 30 months past their surgery date; 67 of these (30.0%) have lived ≥ 30 months and have a Kaplan-Meier (KM)-derived mOS of 46.5 months. 182 patients are ≥ 36 months past surgery; 44 of these (24.2%) have lived ≥ 36 months and have a KM-derived mOS of 88.2 months. A population of extended survivors (n = 100) with mOS of 40.5 months, not explained by known prognostic factors, will be analyzed further. Only 2.1% of ITT patients (n = 7) had a grade 3 or 4 adverse event that was deemed at least possibly related to the vaccine. Overall adverse events with DCVax were comparable to standard therapy alone.

**Conclusions:**

Addition of DCVax-L to standard therapy is feasible and safe in glioblastoma patients, and may extend survival.

*Trial registration* Funded by Northwest Biotherapeutics; Clinicaltrials.gov number: NCT00045968; https://clinicaltrials.gov/ct2/show/NCT00045968?term=NCT00045968&rank=1; initially registered 19 September 2002

## Background

Glioblastoma is the most aggressive primary malignant brain tumor in adults [1]. Standard of care (SOC) consists of surgical resection followed by 6 weeks of daily radiotherapy with concurrent temozolomide, then monthly temozolomide [2]. Median overall survival (mOS) under this SOC is only 15–17 months [2, 3], and ≤ 5% of patients are alive at 5 years [3]. Loco-regional therapy with alternating electric fields has recently shown an increase in median PFS (mPFS) to 6.7 months and mOS to 20.9 months from randomization, respectively [4]. However, there has been no material advance in survival with systemic therapies since the addition of temozolomide 12 years ago, despite investigations with many diverse agents [2, 5–10].

Immunotherapy is an appealing strategy because of the potential ability for immune cells to traffic to and destroy infiltrating tumor cells. Dendritic cells (DCs) are central to the immune system as key regulators of immune tolerance and immunity [11]. For more than a decade, our group and others have been testing active vaccination strategies, such as DCs pulsed with tumor lysates or synthetic peptides to induce antitumor immunity in glioblastoma patients [12, 13]. We have previously demonstrated the effectiveness of DC vaccination in pre-clinical models [14–16], and early stage clinical trials have shown substantial promise [17–19].

In this report, we describe the blinded interim data of the overall ITT patient population enrolled in a Phase 3 randomized, double-blinded, placebo-controlled clinical trial of an autologous tumor lysate-pulsed dendritic cell vaccine (DCVax^®^-L) for newly diagnosed glioblastoma. To date, we have not yet reached sufficient events (i.e., deaths) in this trial to justify unblinding. Nevertheless, since the vast majority (86.4%) of the ITT population received the experimental DC treatment at some point during the trial because of the cross-over study design, analysis of the interim data may provide early insight into the impact of DCVax-L on overall survival. A final analysis of the data obtained in this trial following unblinding will occur once sufficient events of disease progression or death have occurred to fully elucidate patient survival data in the tail of the survival curve.

## Methods

### Study patients

Patients were eligible for this study if they were 18–70 years of age and had newly diagnosed glioblastoma, as determined through central pathology review. Other eligibility criteria included Karnofsky Performance Score (KPS) of ≥ 70 [20], adequate bone marrow, liver, and renal function, life expectancy of ≥ 8 weeks, no other prior malignancy within the last 5 years, no active viral infections, and sufficient resected tumor material to produce the autologous vaccine. Patients were excluded if they already had apparent early disease progression/recurrence or pseudo-progression at the baseline visit, similar to the inclusion/exclusion criteria of other recent trials in glioblastoma [4, 21].

### Study design and treatments

We conducted this study at over 80 sites in 4 countries: the US, Canada, Germany, and the UK. Patient recruitment was initiated in 2007, and was paused from 2009 to 2011 for economic reasons. The midpoint of enrollment was reached in May of 2014, and the final patient was enrolled in November of 2015. The protocol was approved by the required independent ethics committees and institutional review boards. Written consent was obtained from all patients participating in the trial.

All patients underwent surgical resection and 6 weeks of chemoradiotherapy per SOC, prior to enrollment and randomization in the study.

Randomization was performed centrally and was stratified by clinical site and MGMT (O^6^-methylguanine-DNA methyltransferase) gene promoter methylation status, which was determined by a central laboratory. Patients were randomized 2:1 to SOC plus autologous DC vaccine (DCVax-L; n = 232) or SOC plus placebo (n = 99). PBMCs were used as placebo control as these cells are visually indistinguishable from DC and are considered immunologically inactive. Patients in both arms continued to receive monthly adjuvant temozolomide (150–200 mg/m^2^/day × 5 days every 28 days), interspersed with the DC vaccine or placebo treatments administered on Days 0, 10 and 20, then Months 2, 4 and 8, and thereafter at 6-month intervals starting at month 12. Each DCVax-L treatment involved a dose of 2.5 million autologous tumor lysate-pulsed DCs administered intradermally in the upper arm, alternating arms between injection visits.

All patients were allowed to receive DCVax-L following tumor progression/recurrence, as well as other approved treatments per local practice. All parties (investigators, patients and sponsor) remained blinded as to which treatment each patient had received prior to crossover. All patients who chose this option were given the active treatment on a re-start schedule with immunizations at Days 0, 10 and 20, and then months 2, 4 and 8, and every 6 months thereafter beginning with month 12, with Day 0 being the day of the first vaccination post progression. To date, DCVax-L has been shipped for 286 patients (86.4%) in the trial.

Both the study treatment (DCVax-L) and placebo (PBMC) were prepared by Cognate BioServices, Inc. for all patients in the US and Canada, and by Cognate and the Fraunhofer Institute for Cell Therapy together for patients in Europe, during the chemoradiotherapy period before the baseline visit. The production of DCVax-L involved processing the resected tumor tissue into a lysate, and then collection, purification, differentiation, activation and loading of the autologous DCs. In general, approximately 2 g of tumor tissue was needed to produce the full ten doses for the 36-month treatment and follow-up schedule. The vaccine was aliquoted in individual doses and cryopreserved at < 150 °C [22]. The doses were stored centrally, and shipped individually to the clinical trial sites.

### Assessments

Baseline assessments included physical examination, neurological examination, vitals, KPS, MRI of brain with and without contrast, hematology (CBC with differential, platelets), and serum chemistries (calcium, magnesium, SGOT, SGPT, alkaline phosphatase, LDH, total bilirubin, BUN, creatinine, electrolytes, glucose). Blood was collected for serum markers of autoimmune disease (anti-DNA) and immune monitoring, at the baseline visit and at treatment visits throughout the trial. MRI brain scans were performed every 2 months, per SOC, after the baseline MRI until radiological tumor progression. All MRI scans were evaluated centrally by 2 blinded independent radiologists, with adjudication by a third such radiologist if needed.

Adverse events were recorded prospectively according to the National Cancer Institute’s Common Terminology Criteria (version 3.0 NCI CTC), until 2 months after the last study treatment. Patients are followed for OS until death.

### Statistical analyses

The study’s primary endpoint is PFS, and the secondary endpoint is OS. PFS has not yet been evaluated for this publication and will be the subject of later analyses to allow for central, multi-factorial assessment by an expert panel, using criteria currently emerging as appropriate for immune therapy in this patient population where progression can be complex to determine and pseudo-progression is a known confounding phenomenon. Analysis of the blinded interim data on OS of the ITT population (using SAS version 9.4) was performed 34 months after the midpoint of patient enrollment, and 16 months after the last patient was enrolled and randomized.

General descriptive statistics include the number of observed values, mean, standard deviation, median, and range values for continuous measures. For categorical variables, the number and percentage of subjects with a specific level of the variable are reported. For survival analyses, Kaplan–Meier (KM) curves were generated, yielding estimates of median survival times, along with the two-sided confidence intervals (95% CIs) and estimates of survival at specific time points.

## Results

### Study patients

**Fig. 1:**
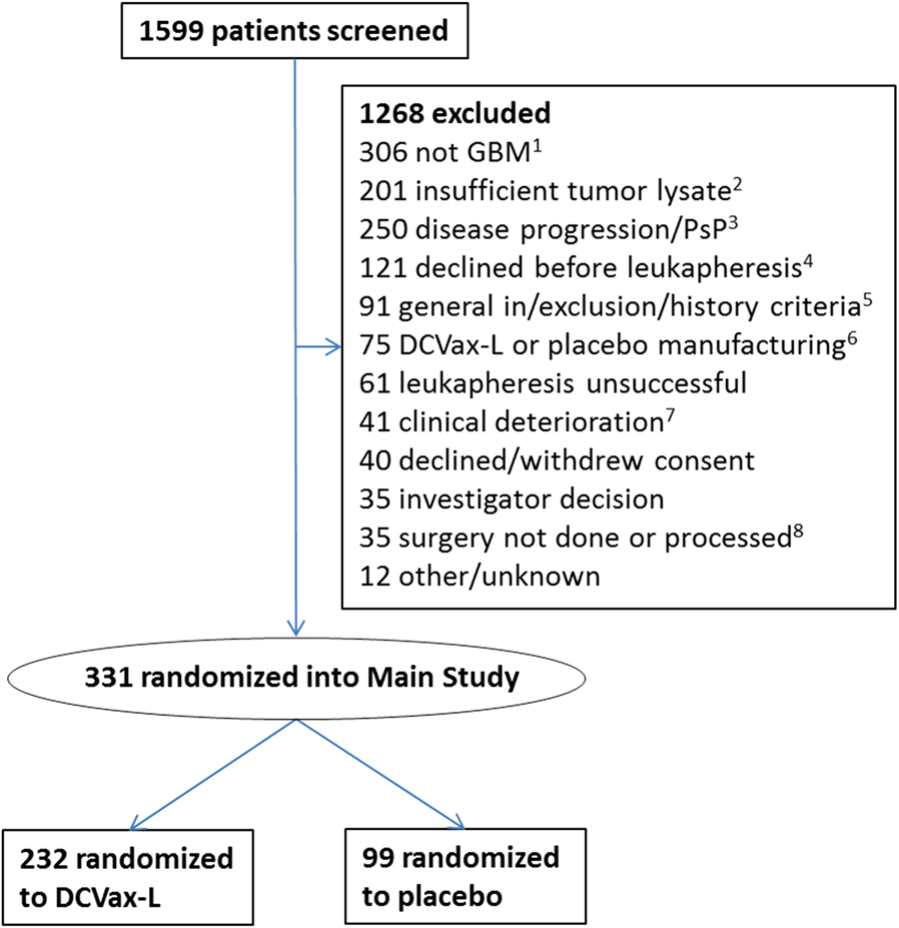
Recruitment, inclusion, and randomization of patients in the study. (1) Patients are screened prior to surgery, so glioblastoma (GBM) determination is made from pathological diagnosis after surgery. (2) Insufficient tumor lysate generated to meet threshold. (3) Progressive disease or pseudo-progression (which are indistinguishable at this point) based on central review of MRI imaging at baseline post-chemoradiation. (4) Patients who consented to tumor donation but then declined participation in trial prior to leukapheresis. (5) Includes deviations from standard chemoradiation protocol, history of prior malignancy, inadequate renal or bone marrow function, etc. (6) Includes drug product failure or insufficient drug or placebo manufactured to meet release criteria. (7) Includes clinical deterioration, declining Karnofsky performance status, or patient deaths. (8) Includes biopsy only, surgery canceled, or tumor tissue not processed after surgery

From July 2007 to November 2015, 331 patients were recruited in the trial, comprising the intent-to-treat (ITT) population. A flow diagram depicting the flow of patients through the screening and enrollment process is provided in Fig. 1. The median time from surgery to randomization was 3.1 months.

The ITT population (n = 331) (Table 1) is similar to other recent glioblastoma trials [4, 21, 24], with 61% males (n = 202) and 39% females (n = 129), with 75.2% of the patients ≥ 50 years of age (range 19–73 years), and median KPS of 90. 63.1% of patients (n = 209) had gross total resection and 36.9% (n = 122) did not. The MGMT gene promoter was methylated in 39.6% of patients (n = 131) and unmethylated in 48.9% (n = 162), with information not available for 11.5% (n = 38; the missing data relates to the early patients enrolled a decade ago). Absolute lymphocyte count (ALC) was > 800 cells/mm^3^ in 48.6% of the patients (n = 161) and was < 800 cells/mm^3^ in 51.4% of patients (n = 170), a characteristic that has been associated with poor prognosis after radiation [23]. Patients with radiographic evidence of disease progression at baseline were excluded, as they have also been excluded in other recent trials for newly diagnosed glioblastoma [4, 21, 24].

**Table 1:** Baseline demographic and clinical characteristics

Variable	n = 331 (100%)
Age (year)	
Mean (SD)	55.33 (10.01)
Median (range)	56 (19, 73)
Sex, n (%)	
Female	129 (39.0)
Male	202 (61.0)
Race, n (%)	
American Indian or Alaska Native	1 (0.3)
Asian	2 (0.6)
Black or African American	7 (2.1)
Hispanic or Latino	16 (4.8)
White	294 (88.8)
Not available^a^	11 (3.3)
KPS at baseline, n (%)	
< 90	97 (29.3)
≥ 90	234 (70.7)
MGMT classification, n (%)	
Methylated	131 (39.6)
Not methylated	162 (48.9)
Not available	38 (11.5)
Lymphocyte group, n (%)	
High	161 (48.6)
Low	170 (51.4)
Surgical status, n (%)	
Partial resection	122 (36.9)
Complete resection	209 (63.1)

^a^Race is in some cases not collected due to institutional policy

Since other treatments were allowed following disease progression, we assessed their usage in this trial. While on study, three patients (1%) had another resection, 103 patients (31%) received bevacizumab, 53 patients (16%) received CCNU and 6 patients (1.8%) were treated with tumor treating fields. In multiple reported studies, neither bevacizumab nor CCNU have been shown to extend survival [9, 25].

### Treatment outcomes

#### ITT population

**Fig. 2:**
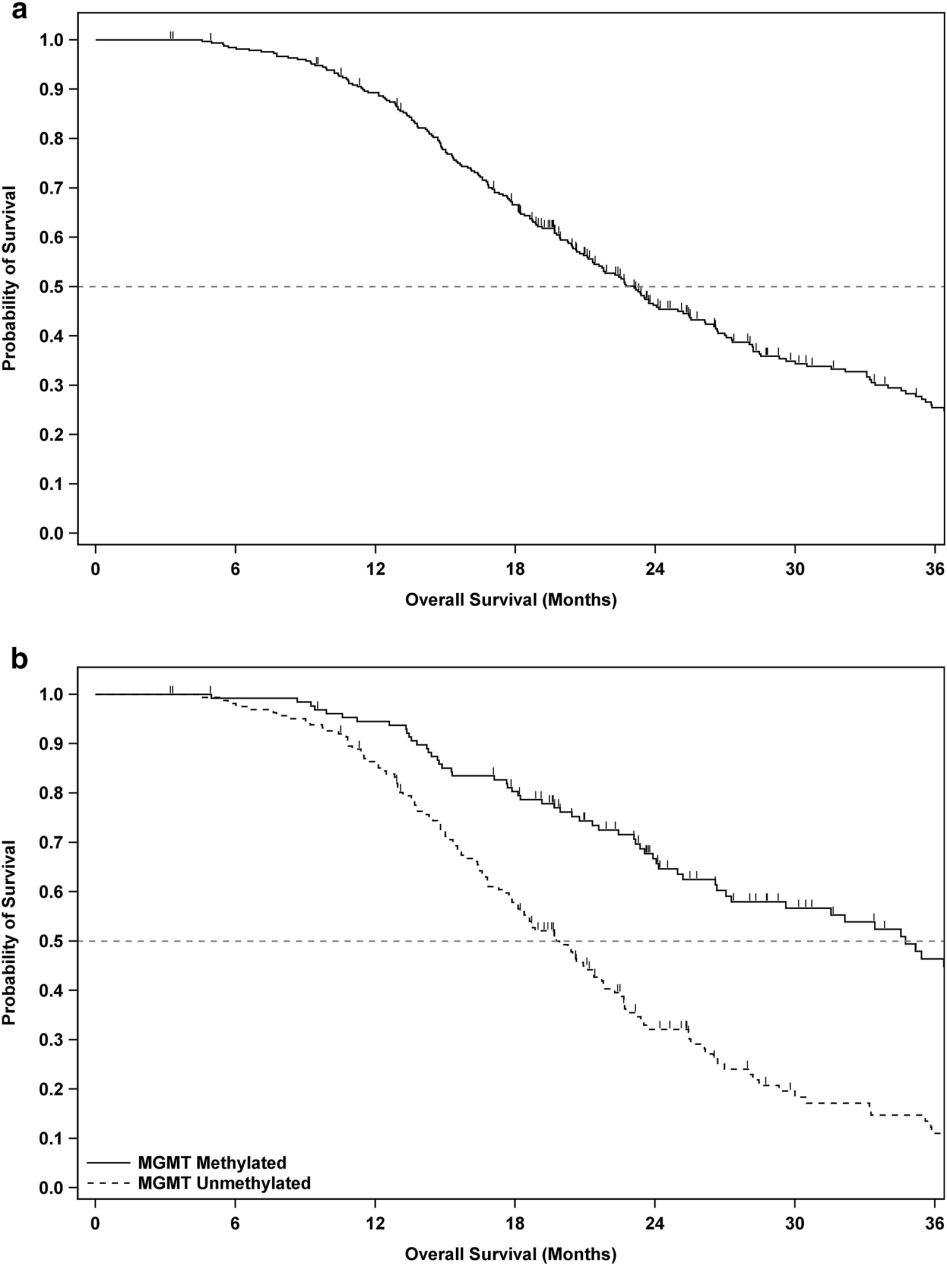
Overall survival curves for patients in the intent-to-treat population. Overall survival analyses of time from date of surgery until death or last follow-up according to the Kaplan–Meier method for all patients in the intent-to-treat (ITT) population (**a**), and the ITT population stratified by MGMT gene promoter methylation status (**b**). Censored patients are annotated by a small vertical line

At the time of this analysis, 108 the 331 patients (32.6%) were still alive. The mOS of the overall ITT population (n = 331) was 23.1 months from the time of surgery (95% CI 21.2–25.4), with 2 and 3-year survival rates of 46.2 and 25.4%, respectively (see Fig. 2a and Table 2). Analysis of patient survival relative to year of enrollment did not reveal a trend over time, nor meaningful differences between years.

**Table 2:** Study endpoints according to molecular genetic and clinical prognostic subgroups

Population	n	Median OS since surgery (months)^a^	Survival at 1 year^b^	Survival at 2 years^b^	Survival at 3 years^b^
Overall	331	23.1 (21.2, 25.4)	89.3% (85.4, 92.2)	46.2% (40.4, 51.8)	25.4% (19.9, 31.3)
MGMT methylated	131	34.7 (27.0, 40.7)	94.5% (88.8, 97.3)	66.7% (57.5, 74.4)	46.4% (35.8, 56.3)
MGMT un-methylated	162	19.8 (17.9, 21.7)	86.4% (80.0, 90.8)	32.1% (24.5, 9.9)	11.0% (5.7, 18.2)
Gross total resection	209	25.4 (21.8, 28.2)	91.8% (87.1, 94.8)	51.2% (43.9, 58.1)	29.9% (22.6, 37.5)
Partial resection	122	21.1 (19.1, 23.1)	85.0% (77.2, 90.2)	37.7% (28.6, 46.7)	18.0% (10.5, 27.1)
KPS at baseline ≥ 90	234	23.7 (21.8, 26.7)	94.0% (90.0, 96.4)	49.2% (42.3, 55.8)	26.6% (19.9, 33.8)
KPS at baseline < 90	97	19.8 (16.6, 23.9)	77.8% (68.0, 84.9)	38.8% (28.5, 49.0)	22.1% (13.4, 32.2)
ALC > 800	161	23.6 (21.7, 28.2)	89.9% (84.0, 93.7)	49.5% (41.1, 57.4)	28.7% (20.6, 37.3)
ALC ≤ 800	170	21.6 (19.9, 25.2)	88.7% (82.8, 92.6)	43.3% (35.4, 50.9)	22.2% (15.0, 30.3)
Age < 50 years	82	26.2 (21.1, 31.5)	92.5% (84.2, 96.6)	51.7% (39.9, 62.3)	28.0% (16.4, 40.8)
Age ≥ 50 years	249	22.4 (20.4, 24.1)	88.2% (83.5, 91.7)	44.4% (37.7, 50.8)	24.6% (18.5, 31.2)

^a^Median overall survival (OS) in months of intent-to-treat (ITT) population, followed by 95% confidence interval in parentheses

^b^Annual rates of percentage surviving in ITT population, followed by 95% confidence interval in parentheses

#### Long tail among ITT population

With immune-based therapies, a key focus is on the tail of the survival curve [26]. Among the ITT patients with a surgery date ≥ 30 months prior to the data collection (n = 223), 30% (n = 67) have lived ≥ 30 months, and their KM-derived mOS estimate is 46.5 months. Among the ITT patients with a surgery date ≥ 36 months prior to the data collection (n = 182), 24.2% (n = 44) have lived ≥ 36 months and their KM-derived mOS estimate is 88.2 months.

#### MGMT status and extent of resection

In patients with methylated MGMT (n = 131), mOS was 34.7 months from surgery (95% CI 27.0–40.7), with 2 and 3-year survival rates of 66.7% and 46.4%, respectively. In patients with unmethylated MGMT (n = 162), mOS was 19.8 months from surgery (95% CI 17.9–21.7), with 2 and 3-year survival rates of 32.1%, and 11.0%, respectively (Fig. 2b and Table 2).

For patients with gross total surgical resection (n = 209), mOS was 25.4 months from surgery (95% CI 21.8–28.2), with 2 and 3-year survival rates of 51.2%, and 29.9%, respectively. For patients with only partial surgical resection (n = 122), mOS from surgery was 21.1 months (95% CI 19.1–23.1), with 2 and 3-year survival rates of 37.7%, and 18.0%, respectively (Table 2).

In patients with both MGMT methylation and gross total resection (n = 83), the mOS was 36.5 months (95% CI 31.5–46.5)—1.8 months longer than the mOS of patients with MGMT methylation and only partial resection (n = 48). In patients with unmethylated MGMT, there was no statistically significant survival advantage with gross total resection compared to only partial resection.

#### Unknown factors: sub-group with extended survival

Approximately 30% of the ITT population (n = 100) showed particularly extended survival, with a KM derived mOS estimate of 40.5 months. This is not fully explained by known prognostic factors, as only some of these patients had positive prognostic factors: only 29% were younger than 50 years of age, 65.9% had methylated MGMT, 71% had a complete resection, and only 8% of these patients had all three positive prognostic factors. These patients will be the subject of extensive further analyses and research.

### Safety and toxicity

Safety and toxicity data were assessed on a blinded basis for all 331 ITT patients. Following SOC chemoradiotherapy, and before any DCVax-L treatment, lymphopenia was the most common adverse event, occurring in approximately 170 patients (51%) [23].

The DCVax-L treatment was well tolerated, with only 7 ITT patients (2.1%) experiencing serious (NCI CTC Grades 3–4) adverse events that were deemed related or possibly related to the DCVax-L treatment. These included cerebral edema in 3 patients (0.9%), seizures in 2 patients (0.6%), nausea in 1 patient (0.3%) and lymph gland infection in 1 patient (0.3%).

The rate of total adverse events with SOC plus DCVax-L was comparable to SOC alone (Table 3). Non-serious adverse events that were considered possibly related to the treatment included injection site reactions, fatigue, low-grade fever and night chills.

**Table 3:** Grades 3–4 treatment-emergent adverse events (TEAE)

System organ class^a^	Number (%) of patients with TEAE (n = 331)
Patients reporting at least one serious TEAE (whether or not related to DC vaccine treatment)	137 (41.1%)
Nervous system disorders	93 (28.1%)
Infections^b^	23 (6.9%)
General disorders and injection site reactions	22 (6.6%)
Respiratory, thoracic and mediastinal disorders	17 (5.1%)
Psychiatric disorders	16 (4.8%)
Gastrointestinal disorders	16 (4.8%)
Injury, poisoning, and procedural complications	12 (3.6%)
Vascular disorders	6 (1.8%)
Musculoskeletal and connective tissue disorders	5 (1.5%)
Neoplasms benign, malignant and unspecified	5 (1.5%)
Hematological disorders	5 (1.5%)
Metabolism and nutrition disorders	3 (0.9%)
Hepatobiliary disorders	2 (0.6%)
Renal and urinary disorders	2 (0.6%)
Cardiac disorders	1 (0.3%)
Ear and labyrinth disorders	1 (0.3%)
Immune system disorders^c^	1 (0.3%)
Reproductive system and breast disorders	1 (0.3%)

^a^Coded per MedDRA 16.0. Patients may have had more than one adverse event, so subcategories do not total

^b^Includes surgical wound infections, meningitis, urinary tract infections, and others

^c^Includes drug hypersensitivity

## Discussion

Although enrollment was completed in 2015, this trial, including both treatments and follow-up, is still ongoing and will remain blinded until sufficient events of disease progression and/or death have occurred to more fully elucidate the tail of the survival curve. To date, due to the crossover design, nearly 90% of the ITT population received DCVax-L at some point in the trial, due to the crossover design.

DCVax-L is administered by intra-dermal injection in the arm, six times in year one and twice per year thereafter. It thereby imposes only a minimal burden on the patient.

In the overall ITT population in this trial, the mOS of 23.1 months from surgery compares favorably with the mOS of 15–17 months from surgery typically achieved with SOC in past studies and clinical practice, as well as with the survival data with SOC treatment in the control arms of other trials in similar patient populations. For example, Weller et al. reported mOS of 17.4 months from randomization in the ITT population [21], and Stupp et al. reported mOS of 16.0 months from randomization in the ITT population [24].

In patients with a methylated MGMT gene promoter, the mOS of 34.7 months from surgery also compares favorably with SOC in past studies as well as with the mOS reported for the control arm SOC treatments in other recent glioblastoma trials in similar patient populations. For example, Stupp et al. reported for their control group an mOS of 21.2 months from randomization in a similar patient population [24]. The increase in survival in MGMT-methylated patients in the DCVax-L trial raises the possibility of a cooperative effect from the combination of temozolomide chemotherapy and the DCVax-L active immune therapy [17].

The mutation status of the IDH1 gene has not yet been investigated for this trial, as this factor was not included in trial designs a decade ago when this trial began. It will be collected and analyzed later, but is unlikely to explain the overall survival results, as the mutation associated with prolonged survival occurs in less than 10% of newly diagnosed glioblastoma patients [27].

Beneficial effects of immune therapies are often observed at later time points, in the tail of the survival curve [26]. Although this Phase 3 trial requires further maturation, a picture is beginning to emerge from the blinded interim data which is consistent with an extended survival tail. For example, among the patients (n = 182) who were ≥ 36 months past their surgery date as of the date of this analysis, 24.2% (n = 44) were alive for ≥ 36 months and have a KM estimated median survival time of 88.2 months. Thus, it appears that patients who survive past certain threshold time points may continue onwards to unusually long survival times, similar to the findings in our prior Phase I/II studies of this DC-based vaccine [17–19]. Further maturation of the trial data is needed to more fully reveal the extent of the long tail of the survival curve.

DCVax-L has shown a benign safety profile in this Phase 3 study, as it has consistently done in prior early stage trials [17, 19], and in a large group of patients treated on a compassionate use basis [28]. The fact that only 7 of the 331 ITT patients (2.1%) experienced any grade 3 or 4 adverse events that were at least possibly related to the treatment makes this DC vaccine an especially well tolerated treatment.

With such a safety profile, this DC vaccine may be administered in a wide range of clinical settings, and can potentially be combined with a wide range of other treatment agents, including immune checkpoint inhibitors and targeted therapies, without resulting in undue toxicities for patients such as have been seen with some other treatment combinations [29, 30]. Further studies to explore such combinations are warranted.

## Conclusions

The addition of DCVax-L autologous dendritic cell vaccine to SOC is feasible and safe. Collectively, the blinded interim survival data suggest that the patients in this Phase 3 trial are living longer than expected. These findings warrant further follow up and analyses.
