# Correction to: First results on survival from a large Phase 3 clinical trial of an autologous dendritic cell vaccine in newly diagnosed glioblastoma

**DOI:** 10.1186/s12967-018-1552-1

**Published:** 2018-06-29

**Authors:** Linda M. Liau, Keyoumars Ashkan, David D. Tran, Jian L. Campian, John E. Trusheim, Charles S. Cobbs, Jason A. Heth, Michael Salacz, Sarah Taylor, Stacy D. D’Andre, Fabio M. Iwamoto, Edward J. Dropcho, Yaron A. Moshel, Kevin A. Walter, Clement P. Pillainayagam, Robert Aiken, Rekha Chaudhary, Samuel A. Goldlust, Daniela A. Bota, Paul Duic, Jai Grewal, Heinrich Elinzano, Steven A. Toms, Kevin O. Lillehei, Tom Mikkelsen, Tobias Walbert, Steven R. Abram, Andrew J. Brenner, Steven Brem, Matthew G. Ewend, Simon Khagi, Jana Portnow, Lyndon J. Kim, William G. Loudon, Reid C. Thompson, David E. Avigan, Karen L. Fink, Francois J. Geoffroy, Scott Lindhorst, Jose Lutzky, Andrew E. Sloan, Gabriele Schackert, Dietmar Krex, Hans-Jorg Meisel, Julian Wu, Raphael P. Davis, Christopher Duma, Arnold B. Etame, David Mathieu, Santosh Kesari, David Piccioni, Manfred Westphal, David S. Baskin, Pamela Z. New, Michel Lacroix, Sven-Axel May, Timothy J. Pluard, Victor Tse, Richard M. Green, John L. Villano, Michael Pearlman, Kevin Petrecca, Michael Schulder, Lynne P. Taylor, Anthony E. Maida, Robert M. Prins, Timothy F. Cloughesy, Paul Mulholland, Marnix L. Bosch

**Affiliations:** 1 University of California Los Angeles (UCLA) David Geffen School of Medicine & Jonsson Comprehensive Cancer Center, Los Angeles, CA USA; 2 King’s College London School of Medical Education, London, UK; 3 University of Florida, Gainesville, FL USA; 4 Washington University, St. Louis, MO USA; 5 Abbott Northwestern Hospital, Minneapolis, MN USA; 6 Swedish Medical Center, Swedish Neuroscience Institute, Seattle, WA USA; 7 University of Michigan Medical School, Ann Arbor, MI USA; 8 University of Kansas Cancer Center, Kansas City, KS USA; 9 Sutter Institute for Medical Research, Sacramento, CA USA; 10 Columbia University Medical Center, New York, NY USA; 11 Indiana University Simon Cancer Center, Indianapolis, IN USA; 12 Overlook Medical Center, Summit, NJ USA; 13 University of Rochester Medical Center, Rochester, NY USA; 14 Rush University Medical Center, Rochester, USA; 15 Rutgers Cancer Institute, New Brunswick, NJ USA; 16 University of Cincinnati Medical Center, Cincinnati, OH USA; 17 Hackensack University Medical Center, Hackensack, NJ USA; 18 UC Irvine Medical Center, Irvine, CA USA; 19 Winthrop-University Hospital, Mineola, NY USA; 20 Rhode Island Hospital, Providence, RI USA; 21 University of Colorado Hospital, Aurora, CO USA; 22 Henry Ford Health System, Detroit, MI USA; 23 St. Thomas Research Institute, Nashville, TN USA; 24 University of Texas Health Science Center, San Antonio, TX USA; 25 University of Pennsylvania Perelman School of Medicine, Philadelphia, PA USA; 26 University of North Carolina, Chapel Hill, NC USA; 27 City of Hope National Medical Center, Duarte, CA USA; 28 Thomas Jefferson University, Philadelphia, PA USA; 29 St. Joseph Hospital, Newport Beach, CA USA; 30 Vanderbilt University, Nashville, TN USA; 31 Beth Israel Deaconess Medical Center, Boston, MA USA; 32 Baylor University Medical Center, Dallas, TX USA; 33 Illinois CancerCare, Peoria, IL USA; 34 Medical University of South Carolina, Charleston, SC USA; 35 Mount Sinai Comprehensive Cancer Center, Miami, FL USA; 36 University Hospitals Case Medical Center, Cleveland, OH USA; 37 University Hospital Carl-Gustav-Carus of Technical University, Dresden, Germany; 38 BG-Klinikum Bergmannstrost, Halle, Germany; 39 Tufts University School of Medicine, Boston, MA USA; 40 Stony Brook University, Stony Brook, NY USA; 41 Hoag Cancer Center, Newport Beach, CA USA; 42 H. Lee Moffit Cancer Center and Research Institute, Tampa, FL USA; 43 CHUSHopital Fleurimont, Sherbrooke University, Sherbrooke, QC Canada; 44 UCSD Health System, UC San Diego, San Diego, CA USA; 45 Neurochirurgische Klinik University Clinic Hamburg-Eppendorf, Hamburg, Germany; 46 Houston Methodist, Houston, TX USA; 47 Geisinger Health System, Danville, PA USA; 48 Klinikum Chemnitz GGMBH, Chemnitz, Germany; 49 Saint Luke’s Cancer Institute, Kansas City, MO USA; 50 Kaiser Permanente Northern California, Redwood City, CA USA; 51 Kaiser Permanente Southern California, Los Angeles, CA USA; 52 University of Kentucky College of Medicine, Lexington, KY USA; 53 Colorado Neurological Institute, Englewood, CO USA; 54 Montreal Neurological Institute and Hospital, McGill University, Montreal, Canada; 55 Northwell Hofstra School of Medicine, Lake Success, NY USA; 56 Department of Neurology, Alvord Brain Tumor Center, University of Washington, Seattle, WA USA; 57 University College Hospitals, London, UK; 58 Northwest Biotherapeutics Inc., Bethesda, MD USA; 59 NYU Winthrop Hospital, Mineola, NY USA

## Correction to: **J Transl Med** (2018) 16:142 10.1186/s12967-018-1507-6

Following publication of the original article [1], the authors reported an error in the spelling of one of the author names. In this Correction the incorrect and correct author names are indicated and the author name has been updated in the original publication. The authors also reported an error in the Methods section of the original article. In this Correction the incorrect and correct versions of the affected sentence are indicated. The original article has not been updated with regards to the error in the Methods section.

Incorrect author name upon publication:

- Tobias Walpert

The correct author name:

- Tobias Walbert

The affected sentence in the Methods section upon publication, with the error marked in bold:

- In general, approximately 2 g of tumor tissue was needed to produce the full ten doses for the 36-month treatment and follow-up schedule. The vaccine was aliquoted in individual doses and cryopreserved at** < 150** **°C** [22].

The corrected sentence, with the corrected temperature marked in bold:

- In general, approximately 2 g of tumor tissue was needed to produce the full ten doses for the 36-month treatment and follow-up schedule. The vaccine was aliquoted in individual doses and cryopreserved at **< − 150** **°C** [22].

The reference which is cited in the above sentence can be reviewed in the original article.

## References

[1] Liau LM, Ashkan K, Tran DD, Campian JL, Trusheim JE, Cobbs CS, Heth JA, Salacz M, Taylor S, D’Andre SD, Iwamoto FM, Dropcho EJ, Moshel YA, Walter KA, Pillainayagam CP, Aiken R, Chaudhary R, Goldlust SA, Bota DA, Duic P, Grewal J, Elinzano H, Toms SA, Lillehei KO, Mikkelsen T, Walbert T, Abram SR, Brenner AJ, Brem S, Ewend MG, Khagi S, Portnow J, Kim LJ, Loudon WG, Thompson RC, Avigan DE, Fink KL, Geofroy FJ, Lindhorst S, Lutzky J, Sloan AE, Schackert G, Krex D, Meisel H-J, Wu J, Davis RP, Duma C, Etame AB, Mathieu D, Kesari S, Piccioni D, Westphal M, Baskin DS, New PZ, Lacroix M, May S-A, Pluard TJ, Victor T, Green RM, Villano JL, Pearlman M, Petrecca K, Schulder M, Taylor LP, Maida AE, Prins RM, Cloughesy TF, Mulholland P, Bosch ML (2018). First results on survival from a large Phase 3 clinical trial of an autologous dendritic cell vaccine in newly diagnosed glioblastoma. J Transl Med..

